# Plant-Assisted Green Synthesis of MgO Nanoparticles as a Sustainable Material for Bone Regeneration: Spectroscopic Properties

**DOI:** 10.3390/ijms25084242

**Published:** 2024-04-11

**Authors:** Edyta Proniewicz, Ajith Mohanavilasam Vijayan, Olga Surma, Aleksandra Szkudlarek, Marcin Molenda

**Affiliations:** 1 Faculty of Foundry Engineering, AGH University of Krakow, 30-059 Krakow, Poland; vijayan@agh.edu.pl; 2 Faculty of Chemistry, Jagiellonian University, Gronostajowa 2, 30-387 Krakow, Poland; olga.surma@doctoral.uj.edu.pl (O.S.); marcin.molenda@uj.edu.pl (M.M.); 3 Academic Centre for Materials and Nanotechnology, AGH University of Krakow, 30-055 Krakow, Poland; aleszkud@agh.edu.pl

**Keywords:** green chemistry synthesis, magnesium oxide nanoparticles, MgONPs, thermogravimetric analysis, TGA, surface analysis, surface-enhanced Raman scattering spectroscopy, SERS, methyl orange, MeO, photocatalytic activity, L-phenylalanine, Phe

## Abstract

This work is devoted to magnesium oxide (MgO) nanoparticles (NPs) for their use as additives for bone implants. Extracts from four different widely used plants, including *Aloe vera*, *Echeveria elegans*, *Sansevieria trifasciata*, and *Sedum morganianum*, were evaluated for their ability to facilitate the “green synthesis” of MgO nanoparticles. The thermal stability and decomposition behavior of the MgONPs were analyzed by thermogravimetric analysis (TGA). Structure characterization was performed by X-ray diffraction (XRD), energy dispersive X-ray spectroscopy (EDS), ultraviolet-visible spectroscopy (UV-Vis), dynamic light scattering (DLS), and Raman scattering spectroscopy (RS). Morphology was studied by scanning electron microscopy (SEM). The photocatalytic activity of MgO nanoparticles was investigated based on the degradation of methyl orange (MeO) using UV-Vis spectroscopy. Surface-enhanced Raman scattering spectroscopy (SERS) was used to monitor the adsorption of L-phenylalanine (L-Phe) on the surface of MgONPs. The calculated enhancement factor (EF) is up to 10^2^ orders of magnitude for MgO. This is the first work showing the SERS spectra of a chemical compound immobilized on the surface of MgO nanoparticles.

## 1. Introduction

Magnesium oxide (MgO) nanoparticles are indispensable in industry (as (photo)catalysts, semiconducting materials, sorbent for contaminants from wastewater, refractory materials, etc.), agriculture, and medicine (as good anticancer, antibacterial, and antioxidant agents) due to their exceptional properties [1,2]. MgO nanoparticles also attract more attention compared to other metal oxide nanoparticles for biological implants [3]. Some reports show that the affinity of bone-forming cells to adhere to MgO is twice that of poly-L-lactic acid (PLLA)-based implants [4]. Their high strength-to-weight ratio, high melting point, lower density, and greater functionality, recycling activity, nontoxicity, and hygroscopic nature make them versatile metal oxide nanoparticles [3,5]. Magnesium is one of the most abundant cations in the human body. It is present in a healthy human at a level of about 0.4 mg/kg and is found primarily in bone [4]. The density of magnesium ranges from 1.74 to 1.84 g/mL, which is almost identical to the density of human bone, which ranges from 1.8 to 2.1 g/mL [6]. Its physical properties, such as elastic modulus and compressive strength, are also much closer to those of human bone [7]. According to the United States Food and Drug Administration (21CFR184.1431), magnesium oxide is among the safest magnesium compounds [8]. Magnesium oxide nanoparticles can bind with human serum albumin (HSA), which promotes cell adhesion and proliferation [9]. This facilitates the adhesion of bone marrow-derived mesenchymal stem cells (BMSCs) to the surface of MgO-based implants [10]. Adhesion is also promoted by activation of the α-subunit of the membrane receptor integrins by magnesium ions [11]. BMSCs play a central role in osseointegration of implants [10]. In vitro studies of seeding of BMSCs and bacterial colonies with MgONPs show that the finest critical concentration of MgO for higher stem cell viability and bactericidal activity is 200 µg/mL [12]. The hygroscopic nature of MgO attributes degradation to the interaction of Mg(OH)_2_ with the stimulated body fluid, forming Mg^2+^ and hydroxide (OH^–^) ions [11]. This increases pH and is one reason for the antibacterial activity of MgO [12]. The increase in Mg^2+^ concentration leads to a decrease in Ca^2+^, due to calcium precipitation, and promotes the formation of an apatite layer [11]. Oxidation or reduction of MgO produces various reactive oxygen species, (ROS) such as peroxides, superoxides, and hydroxyl radicals, which can attack and are lethal to the bacterial cell membrane [13]. These factors have increased research interest in magnesium oxide as a biomaterial for orthopedic implants. In addition, magnesium significantly influences the biological activity of ATP in the body. It is also responsible for the stability of RNA and DNA folding patterns [14]. Numerous enzymes in the body require magnesium as a catalyst [15]. Magnesium-based orthopedic implants would remain in the body for 12 to 18 weeks until the tissue heals and becomes bone [6,16]. When they corrode, they form harmless chlorides that are excreted in the urine [4]. Although magnesium is a suitable candidate for implants, high concentrations can cause muscle paralysis, respiratory distress, and hypotension when serum levels exceed 1.05 mM/L. A serum magnesium level of 6–7 mM/L can lead to heart failure and various other problems [10]. However, the likelihood of this complication is low because the body’s renal function works effectively to excrete excess magnesium through the urine [10]. All of the factors described above prove that magnesium is critically important in our bodies in many ways, making it a suitable candidate for biomedical applications. In addition to its use as an implant, MgO can be used in medicine as an anticancer agent, antibacterial component, molecular biosensor, dental implant, and contrast agent for medical imaging, etc. [17].

MgO nanoparticles can be synthesized by well-known methods such as the sol-gel method, the hydrothermal method, co-precipitation, and microwave-assisted synthesis [3,8,18,19,20]. These methods require careful control of reaction parameters such as pH, temperature, and pressure. The use of toxic chemical reagents and the formation of toxic byproducts during synthesis can compromise the biocompatibility of the synthesized MgO nanoparticles [15]. Introducing a widely accepted technique called “green chemistry” could eliminate the drawbacks of conventional physical and chemical synthesis methods. By definition, these are techniques for developing important products and processes that reduce or eliminate the use and generation of hazardous chemicals. The principles of green chemistry could lead to sustainable research [21]. Green synthesis is supported by either prokaryotic bacteria or eukaryotic fungi and plants [22]. Compared to synthesis using microorganisms, which undergo genetic mutations over time and require more time to execute in the laboratory, plant-mediated synthesis is a simple, one-step process. Phytochemicals can act as reducing agents in the synthesis of nanoparticles [22]. It has already been reported that various plant parts are suitable for green synthesis. In 2015, Sushma et al. vividly illustrated the plant-assisted synthesis of nanoparticles in their work [23]. The properties of the desired product may vary depending on the source of the plant extract [22].

Although metal nanostructures improve the properties of implanted/structural bone tissue, little is known about their long-term biocompatibility and biodistribution after their in vivo release from the scaffold or interaction at the interface between tissue and implant materials. Therefore, one of the greatest challenges in biomaterial science is to understand and characterize the processes that occur at the surface of this interface. Surface-enhanced Raman scattering (SERS) is a very good technique for detecting and characterizing the interaction between cells, tissues, or biofluids and the implant surface, which is important for osseointegration. SERS is ideal for medical diagnostics, in part because it can detect dilute analytes (of concentrations down to 10^–14^ M) and allows the use of fiber-optic probes in the visible or near-infrared spectral range, making it an ideal medical diagnostic tool for evaluating hollow organs and implants.

**Figure 1:**
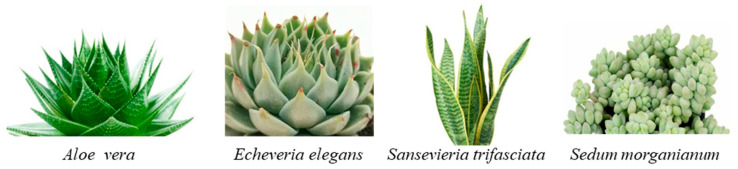
Readily available plants used for the synthesis of MgONPs by the “green chemistry” method.

With this in mind, this work employed a simple and inexpensive method for the biosynthesis of MgO nanoparticles using an extract from several plants, including *Aloe vera*, *Echeveria elegans*, *Sansevieria trifasciata*, and *Sedum morgananum* (Figure 1). The resulting nanoparticles were characterized by molecular spectroscopic methods, including thermogravimetric analysis (TGA), scanning electron microscopy with energy dispersive X-ray spectroscopy (SEM-EDS), dynamic light scattering (DLS), ultraviolet-visible spectroscopy (UV-Vis), X-ray powder diffraction (XRD), and Raman scattering spectroscopy (RS). The ability of MgONPs to photodegrade MeO and the adsorption on their surface monitored by SERS were also determined. For the first time, the SERS spectra of the adsorbate on the MgO nanoparticles as substrate are presented.

## 2. Results and Discussion

### 2.1. Spectroscopic Characterization

**Figure 2:**
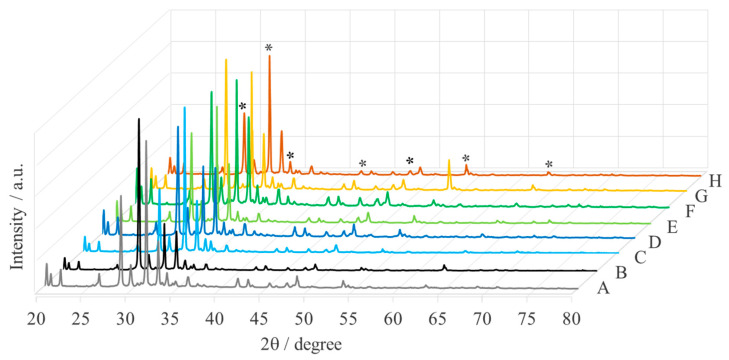
XRD patterns of crude MgONPs from “green chemistry” (A—*Aloe vera*-1, B—*Aloe vera*-2, C—*Echeveria elegans*-1, D—*Echeveria elegans*-2, E—*Sansevieria trifasciata*-1, F—*Sansevieria trifasciata*-2, G—*Sedum morganianum*-1, and H—*Sedum morganianum*-2) (*—diffraction peak from magnesium oxide).

**Figure 3:**
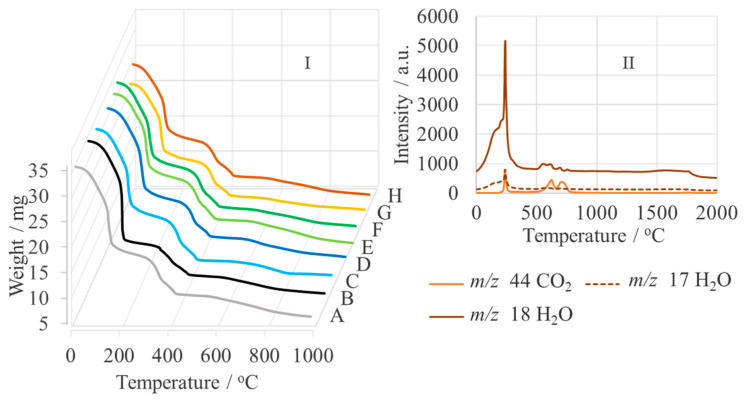
TGA analysis (**I**) of crude MgONPs from “green chemistry” (A—*Aloe vera*-1, B—*Aloe vera*-2, C—*Echeveria elegans*-1, D—*Echeveria elegans*-2, E—*Sansevieria trifasciata*-1, F—*Sansevieria trifasciata*-2, G—*Sedum morganianum*-1, and H—*Sedum morganianum*-2) and QMS analysis (**II**) of MgONPs from *Sedum morganianum*-2.

Figure 2 shows the XRD patterns of crude MgO nanoparticles from “green chemistry”. These patterns contain a series of diffraction peaks, corresponding to the impurities in the samples. To remove the impurities, the samples were calcined, and the calcination temperature was selected based on thermogravimetric analysis (TGA). The thermal properties of MgONPs were studied by monitoring the weight loss, as shown in Figure 3I. Thermal decomposition was observed for all samples in the temperature range of 25–500 °C in three thermal decomposition stages. Decomposition in the first stage occurs in the temperature range of 35–200 °C and causes a weight loss of about 50%. This is due to the loss of water and a small amount of carbon dioxide, which was probably adsorbed (Figure 3II). In the second decomposition stage, in the temperature range of 300–400 °C, a weight loss of about 15% was observed, due to the decomposition of magnesium hydroxide and the oxidation of organic residues, which occurs with the formation of carbon dioxide and water vapor. In the third stage, in the range of 400–450 °C, the weight loss of about 7% is due to the decomposition of carbonate and the oxidation of the remaining organic compounds. Above 500 °C, the weight loss was less than 7% for all samples, indicating stabilization of the crystalline solid phases.

**Figure 4:**
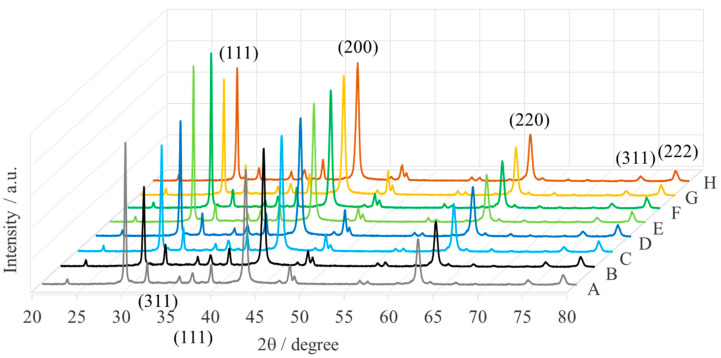
XRD patterns of MgONPs from “green chemistry” calcined at 400 °C (A—*Aloe vera*-1, B—*Aloe vera*-2, C—*Echeveria elegans*-1, D—*Echeveria elegans*-2, E—*Sansevieria trifasciata*-1, F—*Sansevieria trifasciata*-2, G—*Sedum morganianum*-1, and H—*Sedum morganianum*-2).

Figure 4 shows the XRD pattern of MgONPs calcined at a fixed temperature (400 °C). The diffraction peak at about 2θ = 42.8° is due to the (200) crystal plane of MgO. The diffraction peaks at about 36.9°, 62.3°, 74.7°, and 78.6° are consistent with Miller indices (hkl) (111), (220), (311), and (222), respectively, and correspond to JCPDS Card No. 89-7746 cubic crystal structure with lattice parameters of a = 4.2171 Å, S.G. = *Fm*-3*m* (2 2 5), Z = 4 [24,25]. The XRD patterns in Figure 4 also show additional diffractions around 29.35° (strong intensity), 31.80°, 35.44°, 38.98°, 48.41°, and 56.56°. The XRD pattern of MgONPs sintered at 900 °C for 6 h also shows diffractions around 29.35° (111) and 35.44° (311), as in our case [26]. 

The intensity of the XRD reflections of MgONPs indicates that the nanoparticles formed are crystalline, while the narrow diffractions indicate a larger crystallite size. The strong diffractions at 2θ = 42.87° or 29.35° indicate that the MgONPs grow along the (200)-direction (for *Echeveria elegans*-1, *Sedum morganianum-1*, *Aloe vera*-2, *Echeveria elegans*-2, and *Sedum morganianum*-2) or the (111)-direction (for *Aloe vera*-1, *Sansevieria trifasciata*-1, and *Sansevieria trifasciata*-2), which is the preferred orientation of the crystallites. 

The average crystallite size of the MgONPs was calculated using the Debey-Scherrer equation d = kλ/(βcosθ) (where: d is the particle size of the crystal, k is the Sherrer constant (0.94), λ is the X-ray wavelength (1.05406 nm), β is the width of the XRD peak at half-height and is the Bragg diffraction angle) and is on the order of nano-size. Table 1 summarizes the calculated crystallite size of raw MgONPs and MgONPs calcined at 400 °C. It can be clear that the size of MgO nanoparticles from different sources decreases after calcination. This is because calcination removes the water molecules that were previously adsorbed or trapped in the pores or defects of the nanoparticles. The hydrated species formed by the hygroscopic nature of the MgO nanoparticles are desorbed during calcination at lower temperatures [27]. This leads to shrinkage of the crystal lattice to achieve an equilibrium unit cell size [28].

**Figure 5:**
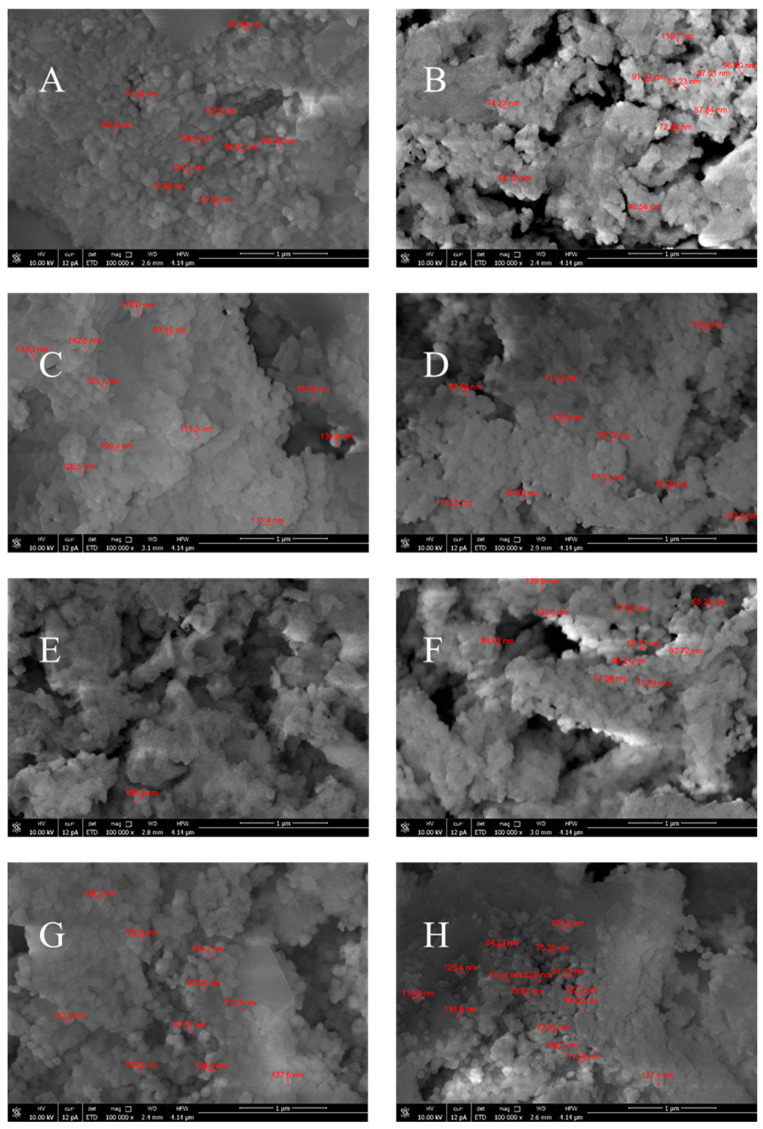
SEM images of MgONPs from “green chemistry” calcined at 400 °C (**A**—*Aloe vera*-1, **B**—*Aloe vera*-2, **C**—*Echeveria elegans*-1, **D**—*Echeveria elegans*-2, **E**—*Sansevieria trifasciata*-1, **F**—*Sansevieria trifasciata*-2, **G**—*Sedum morganianum*-1, and **H**—*Sedum morganianum*-2).

The SEM analysis of the surface morphology of the MgONPs calcined at 400 °C (see Figure 5) shows that the nanoparticles are agglomerated and have a spherical shape with minor variations in size and shape. Table 2 gives an overview of the size of the individual MgO nanoparticles and their average size. As can be seen from this table, the MgONPs that were subjected to an additional water-washing process have a smaller average particle size. Moreover, the uniform distribution of particles over the whole surface can be observed. The aggregation could be due to the interactions and Van der Waals forces between the MgONPs [29]. Researchers also reported that larger grains could be attributed to the Oswald ripening process, with limited porosity and crystallinity [30].

**Figure 6:**
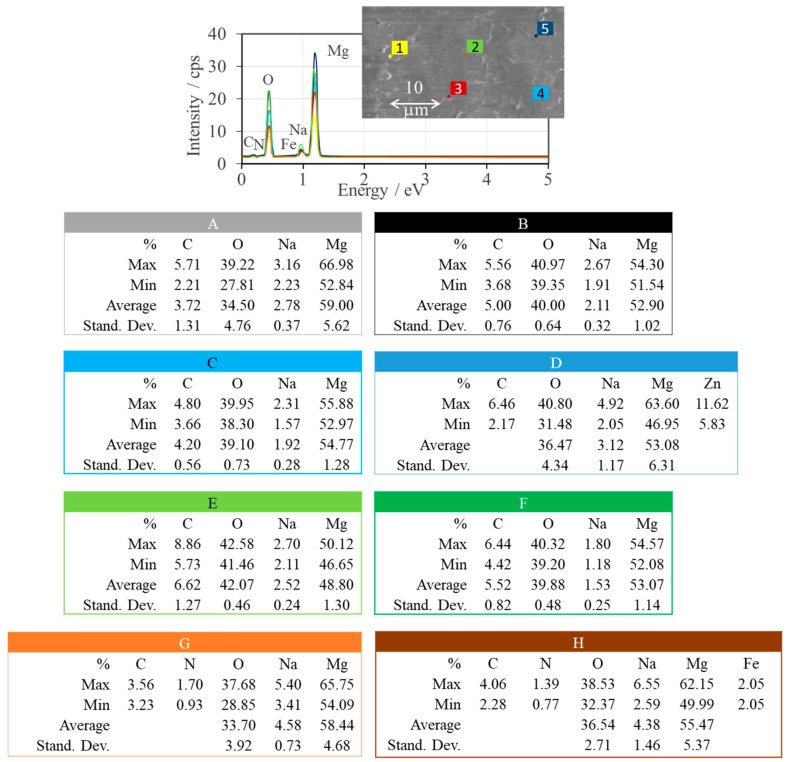
EDS spectra of *Sedum morganianum*-2 collected at 1, 2, …, and 5 measuring points and EDS analysis of MgONPs from “green chemistry” calcined at 400 °C (**A**—*Aloe vera*-1, **B**—*Aloe vera*-2, **C**—*Echeveria elegans*-1, **D**—*Echeveria elegans*-2, **E**—*Sansevieria trifasciata*-1, **F**—*Sansevieria trifasciata*-2, **G**—*Sedum morganianum*-1, and **H**—*Sedum morganianum*-2).

The EDS profile is the additional evidence for the formation of MgONPs. Figure 6 shows EDS chemical analysis for five different probing sites on the surface of sample *Sedum morganianum*-2 and shows the same peaks between 0.5 and 1.5 kV (Mg: K_α_ = 1.27 keV and O: K_α_ = 0.52 keV), indicating the presence of MgO, but the intensity of the corresponding peaks is different [31]. Therefore, qualitative analysis was performed for all samples after averaging the results for each sample. The average content of magnesium (Mg) and oxygen (O) is shown in Figure 6, insets A–H. The small amounts of the other chemical elements, including carbon (C) and sodium (Na), are detected in all samples at about K_α_ = 0.26 keV and K_α_ = 1.05 keV, respectively [31]. The presence of carbon comes from the residual gasses in the SEM chamber, and the sodium comes from the residues of the sodium hydroxide used in the synthesis. No other peaks of other chemical elements were observed in the spectra of *Aloe vera*, *Sansevieria trifasciata*, and *Echeveria elegans*-1, again confirming that the grown MgONPs are pure. Two of the five EDS spectra of *Echeveria elegans*-2 consist of a peak (L_α_ = 1.02 keV) due to the presence of zinc (Zn) (Figure 6D). It should be recalled that one of the steps was omitted in the synthesis of the sample labeled -2 compared to the samples labeled -1: namely, washing the precipitate with distilled water. Considering that zinc oxide nanoparticles (ZnONPs): (1) are obtained in a similar synthesis as MgONPs and (2) are soluble in water [32], it can be assumed that the *Echeveria elegans* sample contained insignificant amounts of ZnONPs that were removed by washing with distilled water. Similarly, trace amounts of iron (Fe, L_α_ = 0.71 keV) were detected in the *Sedum morganianum*-2 sample.

**Figure 7:**
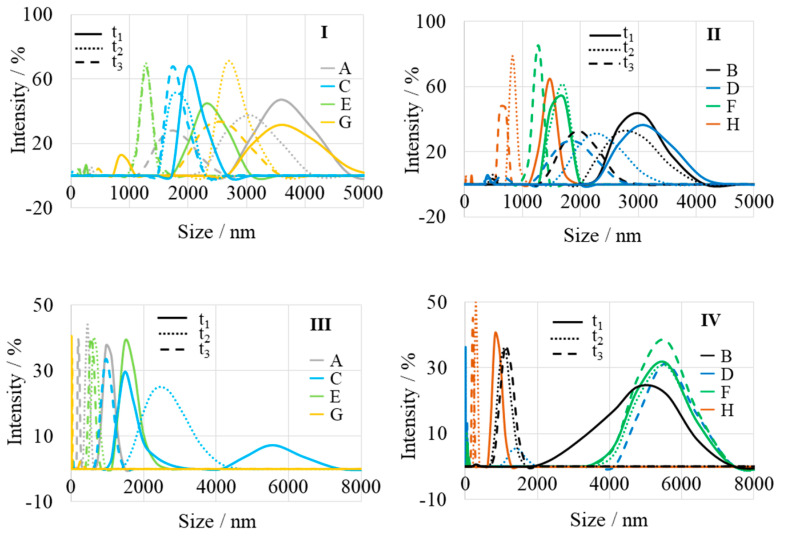
DLS analysis of crude (**I**,**II**) and calcined at 400 °C (**III**,**IV**) MgONPs from “green chemistry” (A—*Aloe vera*-1, B—*Aloe vera*-2, C—*Echeveria elegans*-1, D—*Echeveria elegans*-2, E—*Sansevieria trifasciata*-1, F—*Sansevieria trifasciata*-2, G—*Sedum morganianum*-1, and H—*Sedum morganianum*-2).

Figure 7I–IV shows the distribution of particle size (aggregates) by intensity. All aqueous solutions of MgONPs were sonicated before measurement, but the suspension was unstable and the nanoparticles fell. For this reason, the results measured consecutively (for t_1_, t_2_, and t_3_) differ. Time t_1_ corresponds to the first measurement of the “largest population”. Over time (t_2_ and t_3_), the nanoparticles sink to the bottom and their size decreases. The results shown in Table 3 indicate the estimated average particle sizes. The size of MgONPs from “green chemistry” is larger than that of nanoparticles synthesized by other methods. For example, Wong et al. prepared MgONPs with an average size of 21–122 nm by the Pechini method using ultrasound [33]. Gajengia et al. prepared MgO nanoparticles with an average particle size of 12–22 nm by the microwave method [34]. Kumar et al. prepared MgO nanostructures with a particle size of 20–25 nm by a combustion method [35].

**Figure 8:**
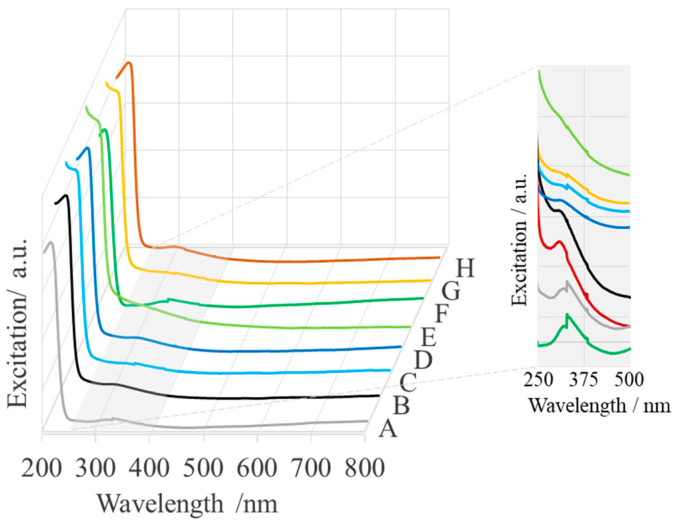
UV-Vis spectra of “green chemistry” MgONPs calcined at 400 °C (A—*Aloe vera*-1, B—*Aloe vera*-2, C—*Echeveria elegans*-1, D—*Echeveria elegans*-2, E—*Sansevieria trifasciata*-1, F—*Sansevieria trifasciata*-2, G—*Sedum morganianum*-1, and H—*Sedum morganianum*-2).

Pure MgO nanoparticles have been reported to exhibit a sharp absorption band at 185 nm, corresponding to the optical excitation of a fivefold coordinated O^2−^ anion [36]. Sackey et al. observed absorption bands at 205 nm (6.04 eV) and 276 nm (4.49 eV) for MgO [30]. These bands were identified with the excitation of quadro- and tri-coordinated O^2−^ anions at the edges and corners of MgO, respectively [37,38,39]. Two different absorptions at 250 and 320 nm were also observed for MgONPs of *Saussurea costus biomasses* [40]. Amina et al. attributed the appearance of the band at wavelengths lower than 300 nm to the presence of tiny particles, while the band at longer wavelengths was attributed to anisotropic nanoparticles [41]. In this work, the characteristic absorption of calcined MgONPs is observed in the wavelength range of 217–230 nm and 313–328 nm (see Figure 8 and Table 2), which is in agreement with other literature results.

The UV-Vis spectrum of MgONPs was further used to calculate the E_g_ values (eV) of the synthesized nanostructures using Planck’s equation (αhν)^2^ = (hν − E_g_), where hν represents the optical energy and absorption coefficient of the material [42]. The calculated band gap energy is 5.71–5.39 eV and 3.96 eV–3.78 eV, respectively (Table 2), which is lower than that of bulk MgO, which has a band gap energy of 7.80 eV [42]. The lower band gap energy can be explained by the presence of 4-coordinated surface anions at the edges of the MgONPs, while the bulk material contains 6-coordinated surface anions [43]. 

**Figure 9:**
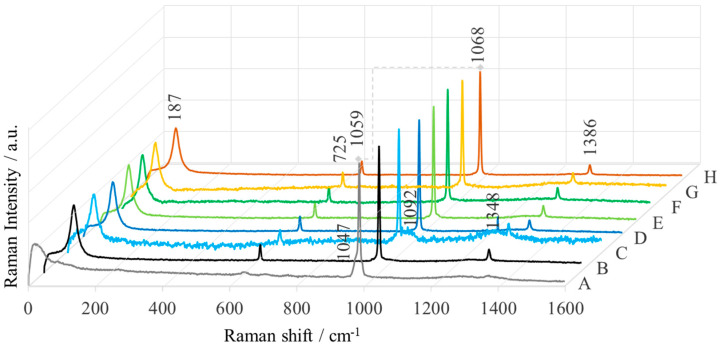
Raman spectra of MgONPs from “green chemistry” after calcination at 400 °C (A—*Aloe vera*-1, B—*Aloe vera*-2, C—*Echeveria elegans*-1, D—*Echeveria elegans*-2, E—*Sansevieria trifasciata*-1, F—*Sansevieria trifasciata*-2, G—*Sedum morganianum*-1, and H—*Sedum morganianum*-2).

Due to its inversion symmetry, MgO as a bulk does not have a Raman spectrum [44]. For micro- and nanocrystals of MgO, the observed Raman bands refer only to the surface information. The Raman spectra of the MgO nanoparticles synthesized by “green chemistry” in this work, recorded in the spectral range of 200−1600 cm^−1^, are shown in Figure 9. These spectra show bands at 187, 725, 1068, and 1386 cm^−1^. The Raman signal at 187 cm^−1^ is absent in the Raman spectrum of MgONPs from *Aloe vera-1*, the band at 1068 cm^−1^ shifts downward in *Aloe vera*-1 (1059 cm^−1^), and the bands at 725 and 1386 cm^−1^ decrease in intensity. Simultaneously with these changes, the appearance of a band at 1048 cm^−1^ is observed for *Aloe vera*-1. In addition, two new broad bands appear at 1092 and 1348 cm^−1^ for *Echeveria elegans*-1. 

Group theory predicts that MgO with a cubic structure has four Raman active modes (A_1g_, E_g_, F_1g_ (silent), and 2F_g_) and two IR active fundamentals (4F_1u_ and F_2u_ (silent)) [45]. For microcrystals of MgO with a particle size of 10–30 nm, Brockelman and coworkers reported Raman active bands at 579, 719, and 1096 cm^−1^ [46]. Ishikawa et al. also showed bands at 280, 446, and 1088 cm^−1^ [47]. The band at 1090 cm^−1^ is assigned to TO-LO modes due to the surface phonon gap, while the D-band is observed below 1500 cm^−1^ (breathing mode) [22,48]. The spectral features below 300 cm^−1^ are attributed to the lattice modes. According to Ishikawa et. al, the weak band at about 280 cm^−1^ is due to a transverse acoustic (TA) phonon (Brillouin scattering) at the zone boundary of the nanostructure [49]. The Raman mode of transverse acoustic (TA) and transverse optical (TO) for MgO particles appears at 742 cm^−1^ [50]. 

### 2.2. Photocatalytic Activity

The application of MgO nanoparticles depends on their morphology, size, shape, and crystalline structure, which affect their surface area, chemical and thermal stability, biocompatibility, wide band gap, low refractive index, good electron transfer ability, surface defects, or the formation of free oxygen species on their surface [51,52]. The ability of MgONPs to dissolve, leading to the toxicity of free metal ions and reactive oxygen species (i.e., hydroxyl radicals (*OH), superoxide ions (O_2_*), hydrogen peroxide (H_2_O_2_), and hydroperoxyl radicals) and alkaline effect, is responsible for their antibacterial properties by causing oxidative stress [53,54,55,56]. A possible mechanism underlying the photocatalytic/antimicrobial effects of nanostructures has been described by Pavlatou and colleagues [55].

**Figure 10:**
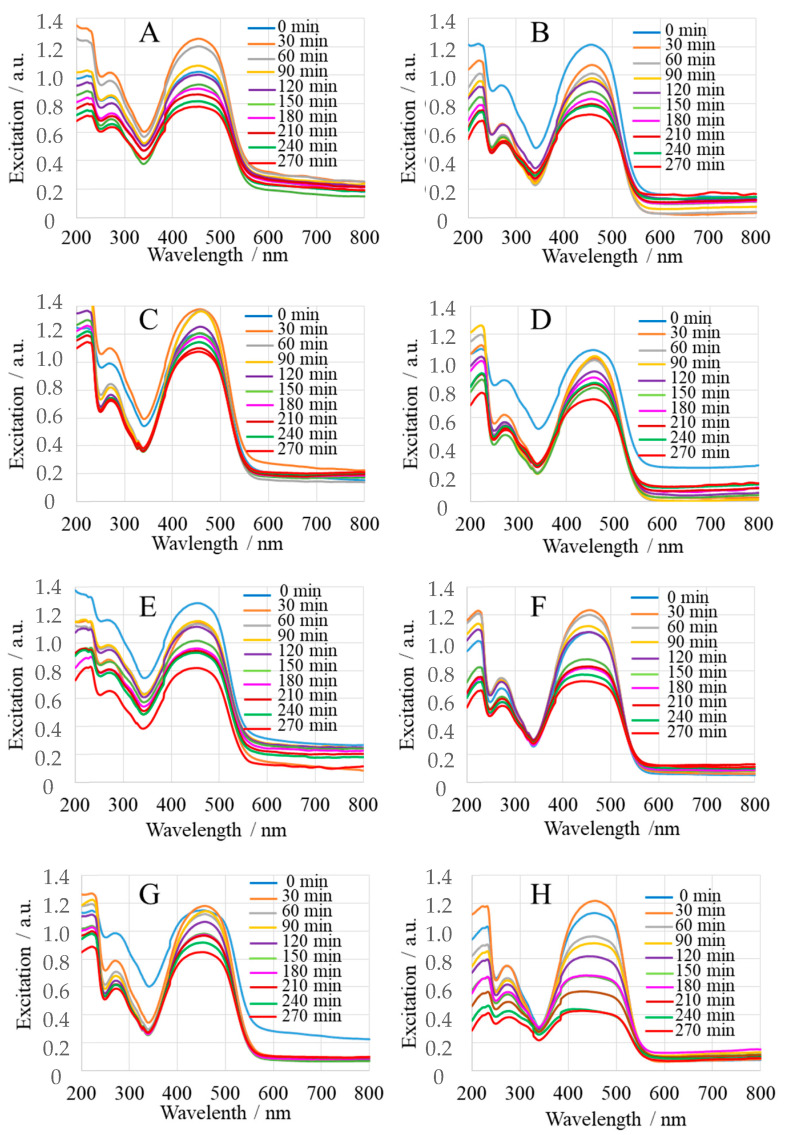
Photodegradation efficiency of MeO with “green chemistry” MgONPs after calcination at 400 °C **A**—*Aloe vera*-1, **B**—*Aloe vera*-2, **C**—*Echeveria elegans*-1, **D**—*Echeveria elegans*-2, **E**—*Sansevieria trifasciata*-1, **F**—*Sansevieria trifasciata*-2, **G**—*Sedum morganianum*-1, and **H**—*Sedum morganianum*-2). Experimental conditions: pH 7.4.

Methyl orange (MeO), a common synthetic azo-organic dye, was used to evaluate the photocatalytic activity of MgO nanoparticles under 365 nm light irradiation. Figure 10A–H shows the time-dependent UV-Vis spectra of the reaction mixture MeO (6 × 10^–8^ mol): MgONPs (10^–5^ mol) after irradiation in the time interval from t_0_ (immediately after mixing) to 270 min after irradiation (t_270_), measured every 30 min. As can be seen, MgONPs from *Sedum morganianum*-2 with a crystallite size of 49 nm are the most effective in photocatalytic degradation of MeO, removing a total of 80% of the dye in 270 min (Figure 10H), whereas MgONPs from *Sedum morganianum*-1 with a crystallite size of 50 nm have a degradation efficiency of 30% (Figure 10G). On the other hand, MgONPs from *Echeveria elegans*-1 (Figure 10C), with the same crystallite size as MgONPs from *Sedum morganianum*-2, have the lowest degradation efficiency of 23% (MgONPs from *Echeveria elegans*-2: degradation efficiency of 33% (Figure 10D)). As shown in Figure 10 and Table 2, the MgONPs subjected to an additional water-washing process (samples-2) exhibited higher degradation efficiency, which is probably related to the fact that water molecules were adsorbed or entrapped in the pores or defects of the nanoparticles. 

The physical adsorption of water molecules only takes place if MgO has surface defects such as edges, steps, corners, and kinks. Costa et al. have summarized some of the results of research on this topic [57]. For example, the authors mentioned studies in which steps are seen as preferred sites for water dissociation, as well as studies that have shown that hydroxylation of steps is thermodynamically favored over physisorption of water [58]. They also mentioned that Goniakowski and Noguer found increased surface reactivity, which they attributed to the high coordination number of OH groups formed by water dissociation. Other authors have observed increased reactivity with increasing surface roughness, with hydroxides forming on the surfaces. It has also been shown that water saturates the oxygen vacancies [59].

Since the MgONPs from *Sedum morganianum*-2 exhibited the highest degradation efficiency, studies on the dependence of photocatalytic activity on MgONPs dose and solution pH were performed for this sample.

**Figure 11:**
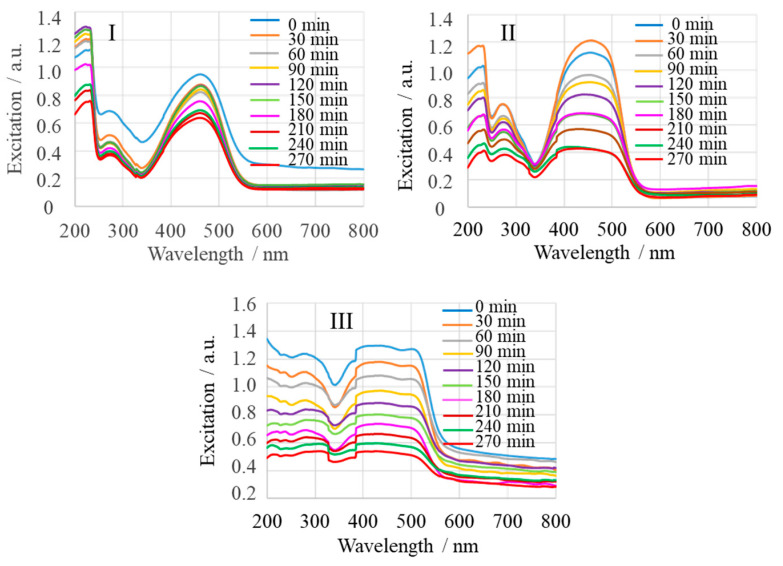
Effect of MgONP (from *Sedum morganianum*-2) dose on photodegradation efficiency of MeO. Experimental conditions: pH 7.4; molar ratio of MeO to MgO of 9 × 10^–3^ (**I**), 6 × 10^–3^ (**II**), and 3 × 10^–3^ (**III**).

MgONPs dose is an important parameter for MeO degradation. Figure 11I–III shows the concentration-dependent UV-Vis spectra of the reaction mixture MgONP:MeO (MgONPs from *Sedum morganianum*-2) after irradiation in the time interval from t_0_ (immediately after mixing) to 270 min of irradiation (t_270_), measured every 30 min. An increase in percent of MeO degradation was obtained with the increase of MgONP dose from a molar ratio of MeO to an MgO of 9 × 10^–3^ (Figure 11I) to 6 × 10^–3^ (Figure 11II) to 3 × 10^–3^ (Figure 11III; UV-Vis analyzer oversaturated during the experiment). Thereafter, the percent of MeO degradation decreased with the increase of MgO nanoparticle dose. This phenomenon can be explained by the fact that the number of active sites increases when the amount of MgONPs is increased. This leads to an increase in the number of adsorbed photons and degraded dye molecules. However, when the molar ratio of MgONPs to MeO was more than 3 × 10^–3^, the percentage of degradation decreased because the large amount of MgONPs blocked the light incidence [60]. 

**Figure 12:**
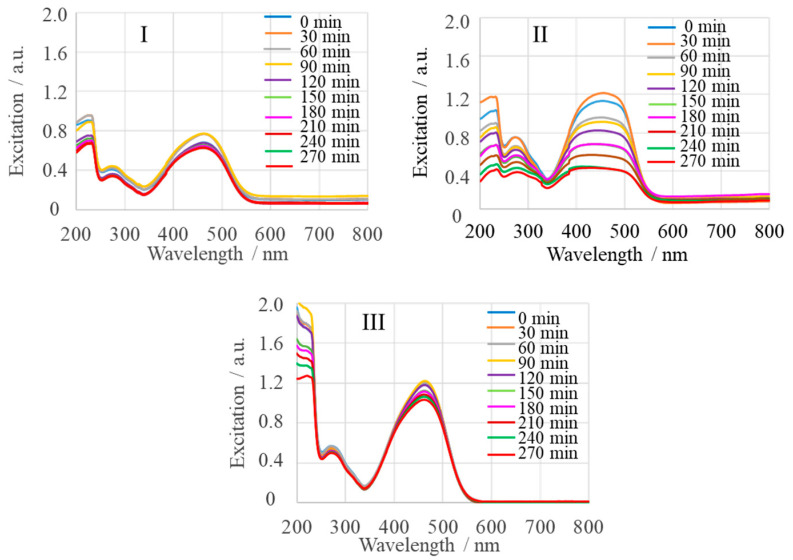
Effect of pH of MgONP (from *Sedum morganianum*-2) solution on photodegradation efficiency of MeO. Experimental conditions: pH of the solution is 5 (**I**), 7.4 (**II**)), and 10 (**III**); molar ratio of MeO to MgO of 6 × 10^–3^.

Figure 12 shows the pH-dependent UV-Vis spectra of the reaction mixture MgONP:MeO (MgONPs from *Sedum morganianum*-2) after irradiation in the time interval from t_0_ (immediately after mixing) to 270 min of irradiation (t_270_), measured every 30 min. Measurements were performed for three different pH solutions: acidic (pH 5) (Figure 12I), neutral (around pH 7.4) (Figure 12II), and alkaline (pH 10) (Figure 12III). The pH 7.4 was chosen because the pH of the human body is in a narrow range between 7.35 and 7.45. As can be seen from Figure 12, an increase in percent of MeO degradation was obtained at pH 7.4, so the photocatalytic activity of various MgONPs was investigated at this pH (Figure 10 and Figure 11).

### 2.3. Adsorption Monitored by SERS

Surface-enhanced Raman scattering (SERS) is a highly sensitive, selective, and relatively fast technique that provides information about surface reactions, interfacial charge transfer, and the architecture of the surface-bound molecule-metal complex. It is used for chemical and biological sensing by enhancing the cross-section of Raman scattering from molecules adsorbed on or near the surface of nanostructured materials [61,62]. The enhancement is achieved by plasmon-mediated electric field enhancement or by chemical enhancement [63]. Noble metals (Ag, Au, and Cu) are the most commonly used plasmonic nanostructured materials in SERS [64]. However, metals other than those in Group 11 (Zn, Fe, Ti, Pt, etc.) are also used, although they are generally considered to have little or no SERS activity [65,66,67,68]. Recently, other materials capable of plasmonically enhanced light-matter interactions, such as Al, In, and Mg, have gained prominence [69,70,71]. However, research results for these metals are very limited. In the case of Mg, only magnesium- or magnesium oxide-doped metals have been reported as SERS substrates for various compounds. For example, the aggregates of nanoMgO and silver have been used to study the SERS activity of some dyes such as methyl blue, malachite green, brilliant green, methyl green, and rhodamine 6G, and nucleobases such as adenine, guanine, cytosine, uracil, and thymine [72]. Doping with Mg and/or Zn improved the SERS property of ZrO_2_ nanoparticles, as demonstrated by the example of 4-4-mercaptobenzoic acid (4-MBA) as a probe molecule [73]. It was reported that the Mg-doped ZnO nanoparticles exhibited stronger SERS activity than pure ZnO when 4-MBA was used as the probe material [74]. In addition, a Mg-Au nanopore substrate was used for SERS detection of lysozyme at low concentrations [75]. Meyer et al. (2011) obtained the SERS spectra of phenylalanine on an aggregate of silver and magnesium chloride [76]. This study is the first to report the SERS enhancement of L-phenylalanine (L-Phe) immobilized on the surface of MgO nanoparticles. This is the first time that SERS spectra have been reported on pure MgO nanoparticles. 

**Figure 13:**
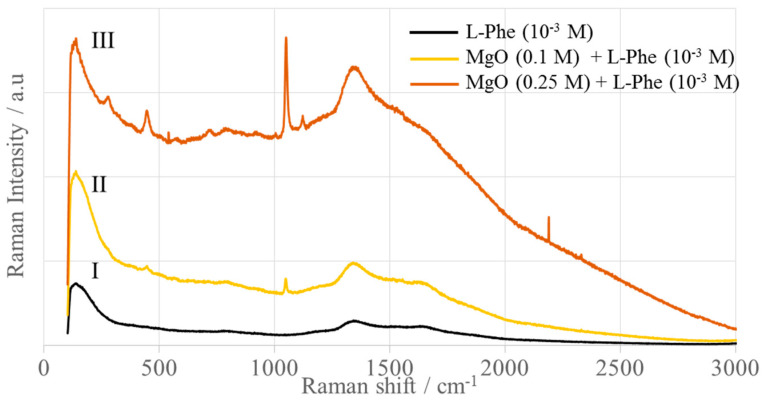
SERS spectra of L-phenylalanine adsorbed on the surface of MgONPs from *Sedum morganianum*-2 calcined at 400 °C.

Figure 13 shows the Raman and SERS spectra of L-phenylalanine (L-Phe) immobilized on the surface of MgONPs from *Sedum morganianum*-2 calcined at 400 °C. As you can see, the L-Phe concentration of 10^–3^ M is too low to obtain a Raman spectrum (Figure 13I). Immobilization of L-phenylalanine with the same concentration on the surface of MgO nanoparticles with a concentration of 0.25 M leads to an enhancement of the bands due to the normal vibrations of this amino acid. They appear at 447 (ν_16a_, ρ_t_(NH_2_)+δ(C–C–N)), 718 (ν_4_), 789 (ν_11_, ρ_r_(C–NH_2_), 1003 (ν_12_), 1043 (ν_18a_), and 1120 cm^–1^ (ρ_t_(NH_2_)) (Figure 13III) [77,78,79]. At a lower concentration of MgONPs (Figure 13II), an enhancement of the L-Phe bands is also observed, but is very weak. This is the first evidence for the SERS activity of MgO nanoparticles.

The L-Phe-bands are enhanced at similar wavenumbers, as observed in the Raman spectrum [77], while their full width is larger in at the half band maximum (FWHM). The intensity ratio of the ν_12_ to the ν_18a_ modes is reversed in the SERS spectrum compared to the Raman spectrum, as well as in the SERS spectrum of the L-phenylalanine phosphate dipeptide L-Phe-L-Ala-CH(OH)-PO_3_H_2_ deposited on the silver surface roughened in oxidation-reduction cycles [79]. This means that the intensity of the ν_12_ modes is very low compared to the intensity of the ν_18a_ mode, which is the most intense band in the spectrum. The FWHM of these SERS signals indicates the interaction of the L-Phe ring with the surface of the MgO nanoparticles, while the low intensity of 1003 cm^–1^ indicates a parallel arrangement of the phenyl ring relative to the surface of the MgONPs [79]. Moreover, the enhancement of the bands due to the vibrations of the –NH_2_ group indicates that this group binds to the MgONPs surface. This result once again confirms the hypothesis that the interaction of the adsorbate with the substrate surface (and thus the geometry of the adsorbate) can be controlled by the choice of the metallic surface. For example, only the aromatic ring of L-phenylalanine is involved in the interaction with the surface of Zn and ZnO nanoparticles [65]. In contrast, on the surface of AgNPs, the carboxyl group is also involved in the interaction with the surface [77], whereas, on the surface of MgO nanoparticles, the amino group is involved in this process. Despite this, coordination of the aromatic ring and the amino and carboxyl groups to the surfaces of AuNPs, α-Fe, and γ-Fe_2_O_3_NPs has been shown [66,80]. 

The enhancement factor (EF) quantifies the effectiveness of the SERS substrate. The electrochemical enhancement factor can be predicted theoretically. The most commonly used definition of EF is EF = (I_SERS_/c_SERS_)/(I_RS_/c_RS_), where I_SERS_ and I_RS_ are the SERS and Raman intensities, respectively, while c_SERS_ and c_RS_ are the analyte concentrations used for SERS and RS measurements, respectively [81]. For the same analyte concentrations, EF is equal to I_SERS_/I_RS_. The calculated EF is up to 10^2^ orders of magnitude for MgO, similar to Zn, Pt, and Fe, compared to 10^6^ orders of magnitude for AgNPs and Au@SiO_2_, 10^5^ orders of magnitude for AuNPs, 10^4^ orders of magnitude for Cu and Ti., and 10^3^ orders of magnitude for ZnONPs, ZnONPs-GS, CuONSs, Cu_2_ONPs, rutile- and anatase-TiO_2_NPs, and γ-Fe_2_O_3_NPs.

## 3. Materials and Methods

### 3.1. Materials

The leaves were obtained from plants: *Aloe vera*, *Echeveria elegans, Sansevieria trifasciata*, and *Sedum morganianum* (Figure 1). Magnesium nitrate (Mg(NO_3_)_2_·6H_2_O), sodium hydroxide (NaOH), and L-phenylalanine (Phe) were purchased from Merck, Poland.

### 3.2. MgO Nanoparticle Synthesis

Freshly picked and washed plant leaves were used to prepare the extract. The extract was prepared in two ways: without deionized water (sample with prefix 1) and with (sample with prefix 2). Briefly, 50 g of leaves were mixed with 50 mL of deionized water and boiled at 90 °C for about 20 min. The obtained extract was cooled and used for the synthesis of magnesium oxide nanoparticles. Then, 10 mL of the freshly prepared leaf extract was mixed with 10 mL of the 1 mM magnesium nitrate solution and stirred for half an hour at room temperature using a magnetic stirrer. Then, 1% NaOH solution was added dropwise until a pH of 12 was reached and was stirred for 2 h at room temperature with a magnetic stirrer until a white precipitate formed on the walls of the vessel. If deionized water was used to prepare the extract, the resulting precipitate was filtered on filter paper, collected on a watch glass, and then washed with ethanol. If deionized water was not used, the resulting precipitate was immediately collected on a watch glass and washed with ethanol. The precipitate was dried at 100 °C for 28 h.

### 3.3. Structure Characterization of MgO Nanoparticles

Thermogravimetric analyses with gas analysis (EGA-TGA) were performed using a Mettler Toledo TGA/SDTA 851e instrument in conjunction with a ThermoStar GSD300T Balzers quadrupole mass spectrometer (QMS). Measurements were performed in air flow (80 cm^3^/min) over a temperature range of 25 to 1000 °C at a heating rate β = 10 °C/min.

The calcination process was carried out in a muffle furnace with controlled air flow. The calcination was a two-step process with increasing temperature, first heated to 350 °C (heating rate of 10 °C/min) and then heated to 600 °C (heating rate of 5 °C/min) for 6 h, followed by cooling.

Scanning electron micrographs (SEM) were taken using a Versa 3D Dual Beam system equipped with ETD and CBS detectors. Simultaneously, the surface of the substrates was analyzed using an energy dispersive X-ray spectrometer with an SDD detector.

The volumetric particle size distribution was determined by dynamic light scattering (DLS) measurements using a Zetasizer Nano ZS analyzer (Malvern Instruments) with an Avalanche photodiode (Q.E. > 50% 633 nm) at 25 °C. Before the measurements, a suspension containing 0.1 wt.% of material was prepared in distilled water and sonicated for 15 min at 20 W (continuous mode) in a Branson SFX250 ultrasonic homogenizer. During sonication, the suspension was cooled to room temperature. To avoid any influence of agglomeration and sedimentation on the results, the DLS measurements were performed within the same time frame, i.e., after 1 min of stabilization. The method used allowed the data to be analyzed within the sample series.

Ultraviolet-visible (UV-Vis) spectra were recorded using a Lambda 25 UV-Vis spectrometer. 

X-ray powder diffractograms (XRD) were recorded using a Bruker D2 Phaser diffractometer. Measurements were made over an angular range of 7° to 80° 2theta with a resolution of 0.02 and a speed of 0.5 s per step. The radiation source in the instrument was a copper lamp, emitting K_α_ = 1.54184 Å.

### 3.4. Photoactivity of MgO Nanoparticles

Photocatalytic studies were performed in a quartz quail initially loaded with 100 µL aqueous 0.1 M MgO nanoparticle solution, 20 µL 3 mM methyl orange solution, and 880 µL H_2_O. Irradiation at 365 nm was performed for up to 270 min and absorbance was monitored using a Lambda 25 UV-Vis spectrophotometer.

Concentration-dependent measurements were made for molar ratios of MgO:MeO of 10:6, 2:6, and 1:6 (MgONPs from *Sedum morganianum*-2).

pH-dependent measurements were made at pH 10, 7.4, and 5 for a mixture with a molar ratio of MgO:MeO of 10:6 (MgONPs from *Sedum morganianum*-2).

### 3.5. Raman and Surface-Enhanced Raman Scattering (SERS) Measurements 

L-phenylalanine (L-Phe) was dissolved in deionized water (0.08 µS/cm) to give a solution with an L-Phe concentration of 10^–3^ mol/L. MgONPs from three reproducible syntheses were used for the study. Two solutions of MgO nanoparticles, with concentrations of 0.25 M and 0.1 M, respectively, were prepared. The MgONP solution was mixed with the L-Phe solution and incubated for 24 h, then the measurements were performed.

Raman and SERS spectra with a spectral resolution of 4 cm^–1^ were recorded using an InVia Raman spectrometer (Renishaw, Poland) equipped with a Leica microscope (50× objective) and an air-cooled CCD detector. A diode laser emitting the 785 nm line was used as the excitation source. The laser power at the output was 10 mW. The typical exposure time for each measurement was 1 accumulation (40 s). SERS spectra were collected at three different locations on three drops of MgONPs/L-Phe (nine spectra in total). No spectral changes were observed that might indicate damage to the sample.

### 3.6. Spectral Analysis

Spectral analysis was performed using a freeware Spectragryph software for optical spectroscopy, version 1.2.16.1, from Dr. Friedrich Menges, Am Dummelsmoos 28, 87561 Oberstdorf, Germany.

## 4. Conclusions

In the present study, we have succeeded in synthesizing MgO nanoparticles using an environmentally friendly plant-based green synthesis method using plant extracts (*Aloe vera*, *Echeveria elegans*, *Sansevieria trifasciata*, and *Sedum morganianum*). 

The crystalline nature of the nanoparticles, which is crucial for the stability and reactivity of MgONPs under physiological conditions, was demonstrated by XRD analysis. TGA analysis demonstrated the stability of the crystalline solid phases, which is an important property because the thermal stability of MgO nanoparticles is responsible for their cytotoxic effect on human cancer cells [26,82], while thermal stability, durability, and biocompatibility make MgO nanoparticles excellent materials for bone and tissue regeneration [83]. The SEM and DLS analyses show the homogeneous morphology (agglomerated spherical nanoparticles) and controlled size distribution (mean particle size of 81–157 nm) required for effective implant integration. EDS results confirm the elemental composition of the samples, thus helping to evaluate their biocompatibility. The Raman spectra confirm the formation of MgONPs. The UV-visible spectra show that the MgONPs from *Sedum morganianum*-2 have the smallest band gap, which allows them to better absorb photons and generate electron-hole pairs that can lead to higher formation of reactive oxygen species (ROS), such as hydroxyl radicals (HO•). Photocatalytic degradation of methyl orange dye under UV light shows that the MgONPs from *Sedum morganianum*-2 also have the highest activity (removing a total of 80% of the dye in 270 min), which is probably related to the adsorption or trapping of water molecules in the pores or defects of the nanoparticles. However, the higher degradation efficiency of MgONPs from *Sedum morganianum*-2 compared to MgO nanoparticles obtained with extracts from other plants may also be related to the fact that only this sample contains trace amounts of iron, most likely in the form of iron oxide, which activates MgO.

The SERS results for L-phenylalanine deposited on the surface of MgONPs from *Sedum morganianum*-2 show that it is possible to monitor interactions between body fluids and additives of implants at the molecular level. This knowledge can help in the future development, improvement, and evaluation of implant materials for better biocompatibility, functionality, and patient outcomes. This is the first work showing the SERS spectra of a chemical compound immobilized on the surface of MgO nanoparticles.

## Figures and Tables

**Table 1:** The crystallite size of crude and, at 400 °C, calcined MgONPs from “green chemistry”.

Sample	Name	Crude MgONPs		MgONPs Calcined at 400 °C	
		Crystallite Size (nm)	2θ Angle	Crystallite Size (nm)	2θ Angle
A	*Aloe vera*-1	65	42.71	49	42.87
B	*Aloe vera*-2	73	42.68	52	42.80
C	*Echeveria elegans*-1	62	42.65	49	42.87
D	*Echeveria elegans*-2	72	42.68	46	42.93
E	*Sansevieria trifasciata*-1	68	42.66	47	42.83
F	*Sansevieria trifasciata*-2	72	42.67	52	42.87
G	*Sedum morganianum*-1	71	42.67	50	42.82
H	*Sedum morganianum*-2	64	42.73	49	42.91

**Table 2:** The particle size of “green chemistry” MgONPs calcined at 400 °C based on SEM and their plasmon resonance, band gap energy, and photodegradation efficiency (UV-Vis).

Sample	Name	MgONPs Calcined at 400 °C						
		Particle Size (nm)	Average Particle Size (nm)	Plasmon Resonance (nm)		Band Gap Energy (eV)		Photodegradation Efficiency (%)
A	*Aloe vera*-1	52–153	94	219	326	5.66	3.80	38
B	*Aloe vera*-2	41–111	81	225	322	5.51	3.85	36
C	*Echeveria elegans*-1	94–176	135	222	319	5.58	3.89	23
D	*Echeveria elegans*-2	57–143	91	224	318	5.54	3.90	33
E	*Sansevieria trifasciata*-1	128–196	154	217	328	5.71	3.78	36
F	*Sansevieria trifasciata*-2	59–133	90	227	327	5.46	3.79	42
G	*Sedum morganianum*-1	94–164	126	225	319	5.51	3.89	30
H	*Sedum morganianum*-2	75–149	110	230	313	5.39	3.96	80

**Table 3:** Estimated average aggregated particle sizes from DLS analysis.

Sample	Name	Crude MgONPs			MgONPs Calcined at 400 °C		
		t_1_	t_2_	t_3_	t_1_	t_2_	t_3_
A	*Aloe vera*-1	3620	3060	1750	1000	455	200
B	*Aloe vera*-2	2980	2800	1970	5080	1150	1080
C	*Echeveria elegans*-1	2020	1810	1740	1480, 5575	2475	970
D	*Echeveria elegans*-2	3080	2285	1850		56, 5540	1400
E	*Sansevieria trifasciata*-1	2330	1280	1275	1520	640	550
F	*Sansevieria trifasciata*-2	1700	1650	1280	5480	5470	5420
G	*Sedum morganianum*-1	3610	2700	2520			280
H	*Sedum morganianum*-2	1480	825	665	860	295	220

## Data Availability

Data are available upon request (proniewi@agh.edu.pl).
